# A systematic review on how environmental sustainability and social responsibility food labels influence consumers’ food choices and purchasing decisions

**DOI:** 10.1017/S1368980026101979

**Published:** 2026-02-11

**Authors:** Jose Izcue, Yasna Palmeiro-Silva

**Affiliations:** 1 MRC Epidemiology Unit, University of Cambridge, Level 3 Institute of Metabolic Science, School of Clinical Medicine, Cambridge CB2 0SL, UK; 2 Center for Health and the Global Environment, Department of Global Health, University of Washington, Seattle, WA 98105, USA; 3 Institute for Global Health, University College London, London, WC1N 1EH, UK

**Keywords:** Food labels, Environmental sustainability, Social responsibility, Nutrition, Behaviour

## Abstract

**Objective:**

In a world under a triple planetary crisis of climate change, biodiversity loss and pollution, this study aimed to evaluate the types, features and impacts of environmental sustainability and social responsibility food labels on consumers’ choices and purchasing decisions.

**Design:**

A systematic review encompassing three electronic databases was conducted. The initial search was conducted in May 2022 and updated in July 2025, identifying 364 studies. After screening, forty-one studies were included. Data were extracted using a standardised form and analysed by topic.

**Setting:**

Studies included were conducted in various consumer and market settings, primarily focusing on packaged food products.

**Participants:**

The studies represented a range of consumers across demographic and geographic contexts, but mostly focused on Western Europe, the US and other high-income countries.

**Results:**

Most studies were experimental (‘choice experiments’) and evaluated purchasing intentions. Environmental sustainability labels generally elicit positive consumer responses, with high preferences for organic and animal welfare claims. Consumers often desire additional information to better understand label meanings. While some evidence supports the influence of environmental sustainability labels on consumer choices, their impact on actual purchasing behaviour remains mixed. Research on social responsibility labels is notably limited.

**Conclusions:**

There is insufficient evidence to determine the real-world impacts of environmental sustainability and social responsibility labels on food choices. Future studies could focus on purchasing behaviours in real-life consumer interactions with labels, the impacts of the exposure to varying levels of information and a potential integration of domains. Given pressing social and environmental challenges, integrative strategies are required to develop labels that effectively guide consumers toward healthier, sustainable, and socially responsible food options.

Over the past century, the *green revolution* contributed to major gains in nutrition, food security and poverty alleviation, by expanding our food systems – food production, distribution and storage^(1)^. However, this process contributed to three major concurrent challenges. Firstly, new public health challenges emerged, including unhealthy dietary patterns and overconsumption of ultra-processed and animal-based products which have largely contributed to non-communicable diseases^(2)^. Secondly, current food systems exert an unsustainable pressure on the planet, including being responsible for approximately 34 % of greenhouse gas (GHG) emissions and 70 % of freshwater withdrawals globally, contributing to climate change and water scarcity. Moreover, agriculture now stands as the primary driver of land-use change and biodiversity decline^(3)^. Thirdly, social inequities in food systems affect millions of smallholder farmers, especially in low- and middle-income countries, who sometimes face unfair trading conditions and poverty despite producing much of the world’s food^(4)^.

These interconnected and complex challenges require a multilevel and multi-sectoral transformation of our food systems. Policies aimed at influencing consumer behaviour and product reformulation have increasingly been implemented in various contexts^(5)^. Food labels are one of such policies and are designed to inform consumers about the implications of food consumption and encourage healthier and better choices. In response to the aforementioned challenges, food labelling has evolved to encompass aspects beyond just nutrition^(6)^, including information on environmental sustainability, fair trade and animal welfare.

Nutrition food labels inform about the ingredients of packaged foods and help people follow dietary guidelines^(7)^. They emerged as back-of-pack nutrition information; however, their effectiveness has been questioned as these are rarely used by consumers^(8)^. Front-of-pack (FOP) nutrition labels, which convey nutrition information on the front of food packaging in a simple way, are now commonly and effectively used to inform consumers about healthier food choices^(9)^. FOP nutrition labels with interpretational aids (i.e. symbols, colours or letters) appear to be slightly more effective in driving consumers toward healthier choices^(10)^ than non-interpretative labels. Some well-known interpretative FOP labels are the UK’s *traffic lights* system, the French Nutri-Score, the Australian *Health Star Rating* system and certain warning labels such as the system implemented in Chile^(11)^.

In parallel, but at a slower pace, environmental sustainability labels have emerged. Although there is no universal definition, environmental sustainability food labels generally inform about the impact of foods on the natural environment. In recent years, several environmental sustainability labelling schemes have been developed^(12)^, but their success in promoting ‘environmentally friendly’ food choices has been less studied^(13)^. These schemes can be classified in various ways, including the International Organisation for Standardisation typologies, such as Type I (eco-label), Type II (self-declared environmental claims) and Type III (environmental product declarations)^(14)^. Examples include the French *Eco-score*, the *Carbon footprint*, the UK’s *Eco impact label* and various organic labels. Whether organic labels should be classified as pertaining to environmental sustainability has been debated^(12)^, but since the concept is commonly understood as promoting environmental protection, organic labels are considered as environmental sustainability labels in this work.

Social responsibility food labels, although not well-defined, can be understood as labels that *‘account for community investment, human rights and employee relations, environmental practices, and ethical conduct’* ^(15)^. These labels usually provide added value to commodities produced by small-scale farmers in low- and middle-income countries. Well-known certification schemes include *Fair Trade*, *Rainforest Alliance* and the *UTZ certification.* While some studies indicate that consumers are Willingness to Pay (WTP) a premium for *Fair Trade* food products^(16)^, only a limited number of studies show a positive impact on real-life purchases and consumer choices^(17)^.

The complexity of the challenges and the often siloed approach to different types of labels has spurred growing interest in developing an integrated food label^(18)^. We might call this a ‘planetary health food label’, that communicates a product’s overall impact across health, environmental and social dimensions. This label can, in theory, empower consumers to make choices that are better for both people and the planet. However, a significant barrier to designing such a comprehensive label is the disparate state of the evidence. While the science behind nutrition labelling is mature, a clear understanding of what makes environmental sustainability and social responsibility labels effective – or ineffective – is lacking. Before an integrated label can be considered, it is crucial to first systematically map the existing evidence for its less-understood components. Therefore, this study aimed to systematically review the evidence on the diversity, features and impacts of environmental sustainability and social responsibility FOP food labels on consumers’ choices and purchasing decisions. Based on the results, we briefly discuss the potential of an integrated food label that aims to inform about the nutrition, environmental sustainability and social responsibility aspects of food products.

## Methods

### Sources and search strategy

We conducted a systematic literature review to evaluate the features and impacts of environmental sustainability and social responsibility labels on consumers’ choices and purchases. We did not include nutrition food labels as they have been extensively analysed^(19)^. The main search was conducted in May 2022 in three multidisciplinary databases: Medline, Web of Science (Core Collection) and Scopus. An updated search was conducted in July 2025 to capture the most recent literature.

The overall search strategy was as follows: ((‘food label*’ OR ‘nutrient* profiling system*’ OR ‘food score*’ OR ‘front of pack nutrition label*’ OR ‘food metric*’) AND (‘impact*’ OR ‘effective*’ OR ‘influence*’) AND [(sustainab* OR ‘land-use change*’ OR ‘water use’ OR ‘greenhouse gas*’ OR ‘environmental footprint’ OR ‘ecological footprint’ OR ‘food footprint’) OR (‘social justice’ OR ‘social responsibility’ OR ‘fairness’ OR ‘fairtrade’ OR ‘equity’ OR ‘livelihood*’)]). Specific keywords are presented in Table A1 in appendix. The keywords were defined as a result of a pre-process analysis of reading-related literature and identifying the most relevant terms, avoiding an ‘explosion’ of irrelevant articles.

To capture the most current evidence, articles included had to be published from 2000 to 2025, and no language restrictions were applied.

### Study selection and data extraction

All identified references were uploaded to *Ryyan* platform (https://www.rayyan.ai/), where duplicates were removed. Titles and abstracts were screened by one researcher, and then two researchers independently reviewed the full texts based on the eligibility criteria (Table 1). Disagreements in the inclusion/exclusion of articles were discussed between the two researchers, which happened with two studies.

**Table 1.** List of inclusion and exclusion criteria

Articles were included if they met the following inclusion criteria:	Articles were excluded if they met any of the following exclusion criteria:
Studies focusing on environmental sustainability food labels or sustainable food profiling models, OR Studies focusing on social responsibility (and its equivalent terms) in the context of food labels or food profiling systems, OR Articles that assess nutrient profiling systems or health-focused food labels, together with either environmental sustainability or social justice dimensions, AND Interventions and studies looking at environmental sustainability or social responsibility labels’ effectiveness, assessed by willingness to pay, purchasing intentions, behaviour change, consumers’ awareness or other measures	Studies not focusing on food labels Studies only focusing on the design aspects of food labels Articles focusing on food labels in the context of nutrition only Animal studies Articles ranking menus or meals, but not individual food items Studies not assessing the impacts or effectiveness of labels in any way

A form was developed to extract data, which included information on country, research design and article type, target population, type of label assessed (i.e. nutrition, environmental sustainability or social responsibility), type of food assessed, outcome studied and main findings. Animal welfare studies were considered and classified as pertaining to environmental sustainability, following the ethical and social value consumers and institutions across the world currently place on this dimension^(20)^.

An internal working protocol was developed prior to conducting the study; however, it was not published elsewhere.

### Critical appraisal

Because of the multidisciplinary nature of this review, a variety of methodologies and methods were anticipated. Therefore, the QuADS tool was used to critically appraise the selected literature, which is used in systematic reviews of mixed or multi-method studies^(21)^. All full texts included in the review were appraised independently by two researchers (JIG and YPS) to characterise the literature rather than select them for inclusion/exclusion and limit the potential findings. Studies that did not meet the inclusion criteria were not considered for critical appraisal.

## Results

**Figure 1.**
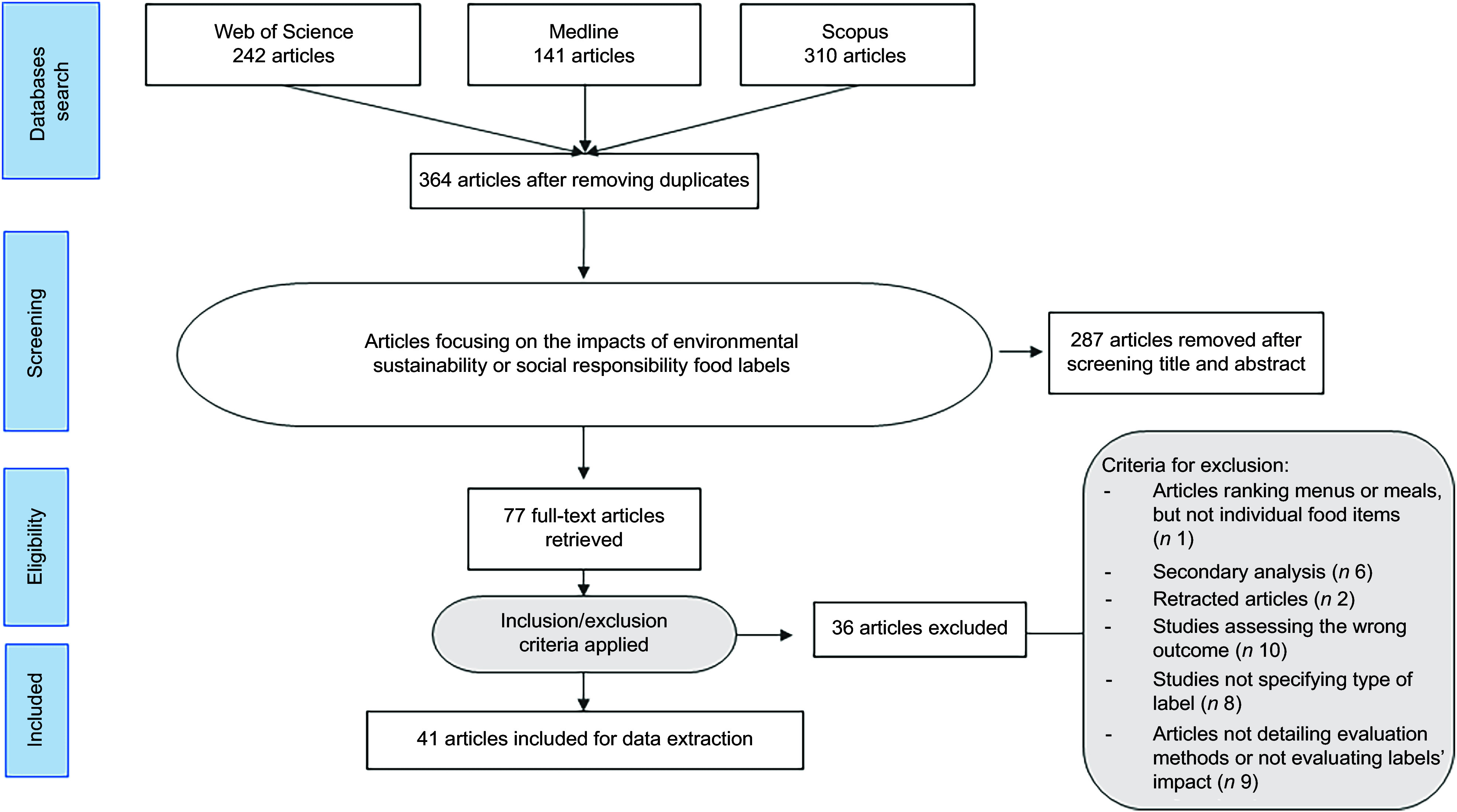
PRISMA flowchart.

A total of 369 articles were identified in May 2022 and 324 in July 2025. Of these, 184 and 180 articles were retrieved after removing duplicates, respectively. After screening titles and abstracts, seventy-seven articles were included for full-text analysis, of which forty-one studies were included for data extraction (twenty-six from the original search in 2022 and fifteen from the update in 2025) (Figure 1).

Most studies were conducted in European countries (*n* 27)^(22–48)^, in the USA (*n* 7)^(49–55)^. Three studies included more than one country^(56–58)^, and one study was from each: China^(56)^, Chile^(57)^, Malaysia^(58)^ and Vietnam^(59)^.

Methodologically, studies varied. Choice experiments (*n* 17)^(23,24,32,34,37,39–41,43,44,46,47,49,50,52,60,61)^ and online questionnaires or surveys (*n* 10)^(25,28,30,35,36,45,48,57,58,62)^ were the most common study designs, with some studies conducting randomised trials and other experimental designs (*n* 11)^(27,29,33,38,42,51,54–56,63,64)^, qualitative and mixed approaches (*n* 3)^(22,26,31)^. Sampling and sample sizes also varied, commonly including online panel samples ranging from ∼100 to ∼3000 participants and generally quoting (or stratifying) for gender, age (> 18 years), education and region of living^(38,52)^. Non-panel studies generally included purposive samples, with unbalanced characteristics of participants (e.g. greater representation of women, people with higher education degree or socioeconomic status)^(33,41,42,58)^.

The food products evaluated included common types of meat (beef, pork, poultry), seafood, dairy, nuts, grains and processed foods. Some studies evaluated an individual product while others included multiple items, but no food group or product predominated over the others. Table 2 summarises the key findings of included articles and describes main characteristics of each study.

**Table 2.** Summary of main characteristics of articles included in the review

First author	Country	Design	Population	Features of label assessed	Type of food assessed	Outcome	Main findings
Apostolidis, C. *et al* (2016)^(23)^	UK	Choice experiment employing a survey (on-site questionnaire in supermarkets)	UK adult consumers (represented by 200 ‘meat eaters’, 200 ‘meat reducers’ and 200 ‘vegetarians’)	Multiple labels on minced meat or plant-based alternative: traffic light for fat, organic logo, GMO logo, carbon footprint logo, EU *v*. non-EU agriculture (local *v*. non-local agriculture)	Meat (red and white: beef, pork, poultry)	Consumers’ preferences (for a given label)	Type of meat, price and fat content labels had the largest impact on consumer choices. The impact of carbon footprint, method of production, origin and brand labels vary across consumer groups. Vegetarian consumers were less concerned about the origin of their food; however, they are still more likely to choose domestically produced food products than imported products. The carbon footprint label had a moderate impact on the choices of vegetarians and meat eaters.
Arrazat *et al.* (2023)^(38)^	France	Randomised controlled trial in a virtual reality supermarket	132 adults responsible for grocery shopping	A novel traffic light (A-E) environmental label based on EF single score	Meal components (e.g. lentils, ham) and ready-to-eat meals (e.g. lasagne)	Environmental impact of food choices (EF single score); meal types selected	The label significantly reduced the environmental impact of food choices, partly by encouraging a shift from meat-based to vegetarian meals, at no extra nutritional or financial cost.
Banovic, M., *et al.* (2019)^(24)^	France, Germany, Italy, Spain and the UK	Discrete choice experiment	EU and UK adult fish consumers responsible for buying food for the household (5 countries)	Sustainability: Produced in its own country label; ASC label. Nutrition: Health and nutrition claim logo.	Fish (aquaculture) with different preserving methods: fresh (chilled), canned and smoked products.	Consumers’ preferences	‘Country of origin’ label (‘produced in own country’) together with ASC eco-label functions better than the health and nutrition claims as driver of choice for aquaculture fish products. Results further point to the existence of different segments of ‘nutrition conscious’, ‘ethnocentric’, ‘price conscious’ and ‘eco-conscious’ consumers.
Berden & Hung (2025)^(39)^	Belgium	Cross-sectional online survey with an information experiment	607 Belgian consumers	Eco-score, presented alongside the Nutri-Score	Twenty-five pre-packaged food products (e.g. lasagne)	Accuracy in assessing environmental impact; intention to use the Eco-score	The Eco-score improved accuracy in environmental impact assessment, but its effect was less pronounced for products with conflicting Eco- and Nutri-Scores, suggesting cognitive dissonance.
Büttner, V., *et al* (2024)^(40)^	Germany	Online questionnaire with a between-subject experimental design	1010 consumers (representative sample for Germany)	Eco-score (A, C and E) *v*. no label	Packaged foods: spaghetti, strawberry yoghurt and crisps	Perceived environmental friendliness, expected tastiness, perceived healthiness and purchase intention	A negative Eco-score (E) created a negative ‘halo effect’, making the product seem less tasty and healthy, which in turn lowered purchase intention.
Carlsson, F., *et al* (2021)^(25)^	Sweden	Stated preference survey	Consumers who regularly buy ready-made meals containing meat, such as lasagne, meatballs and pizza.	Sustainability: Traffic light multilevel label displaying antibiotic use, animal care, climate impact and healthiness. Three versions: coloured traffic light, greyscale circles design, and pure information (text) with no logo. All had 3 levels from low impact to higher impact.	Frozen meat lasagne	Willingness to pay	The red colour in traffic lights strengthened respondents’ preferences for avoiding the worst level of a collective attribute such as climate impact or antibiotics use, while the green colour strengthened preferences for healthiness. On average, the price premiums for a green label compared with a red label is 52 % for healthiness, 64 % for both animal welfare and antibiotics and 20 % for climate impact.
Carlsson, F., *et al* (2022)^(41)^	Sweden	Stated preference survey (choice experiment)	1471 consumers who regularly buy ready-made meat meals	Traffic light-style labels for health, antibiotics use, climate impact and animal care	Frozen lasagne (meat *v*. soy-based)	Choice between meat and meat substitute; Willingness to pay (WTP)	Labels concerning animal care and antibiotics use on meat were most important to consumers. Climate and health labels on the meat substitute increased the likelihood of consumers switching to it.
Carrero, I., *et al* (2021)^(26)^	Spain	Grounded theory – focus groups (qualitative study)	Adults representing mainstream consumers	Sustainability: Carbon (greenhouse gas) emissions label designed for the study. Various designs ranging from warning (red) to ‘positive’ (green) labels	N/A	Label noticeability and use	This study explored the attention-grabbing features of a carbon label. Here, carbon labels were noticed when they were perceived as a cue of hazard. Hence, carbon labels could be designed as warning labels.
Costanigro, M., *et al* (2016)^(49)^	USA	Best–worst choice experiment using a quantitative scale.	Adult consumers in the US (represented in the study by administrative staff of a large university in Colorado)	Sustainability: Organic; Colorado Proud; rBST-free; Validus. The perceptual profile of the four labels was assessed for nine dimensions of sustainability and social justice: animal welfare, energy consumption, water consumption, air pollution, community involvement, employee opportunities, local operations, waste management and sustainable agricultural practices.	Cow’s milk	Willingness to pay (WTP) (for a gallon of milk with the given label *v*. the one without it); Also, ranking of importance of corporate social responsibility areas to improve	Consumers are Willingness to Pay a premium for the labelled products. The strongest result relates to animal welfare: nearly all participants placed animal welfare at the top of their priority list, and they were Willingness to Pay more to sponsor it. Sustainable agricultural practices rank second in the list of consumer priorities, but only one out of the ten models (organic) detects a positive, significant correlation between improved agricultural practices and WTP premium for the label. In general, consumers perceive labels, especially the organic, as indicating many more qualities than they actually do for food products.
De Bauw, M., *et al* (2022)^(27)^	Belgium	RCT (mock e-grocery setting)	Belgian household food decision makers	Nutrition: Nutri-Score. Sustainability: Eco-score.	Multiple foods available at a ‘regular supermarket’	Change in food choices (labels’ impact on product choice)	Displaying Nutri-Score and Eco-score at product level led to improved nutritional qualities of food choices, while the environmental impact was not reduced. Respondents exposed to the scores report to have relied significantly more on both of them, compared to those who were not. Therefore, this discrepancy could not be explained by stated levels of attention being paid to the scores.
De Bauw *et al.* (2024)^(42)^	Spain	RCT in a simulated fishmonger store	Fish consumers	Enviroscore (B, C, D environmental impact levels), with or without supporting information	Seabass products (unprocessed and filleted/packaged)	Product choice (based on Enviroscore level), decision time and stated attention toward various attributes (sustainability, price, origin, health, appearance and quality)	The Enviroscore increased selection of lower-impact fish by 16 %, but only when no supporting information was provided, suggesting the effect is heuristic and unconscious rather than deliberative.
De Marchi, E., *et al* (2016)^(50)^	USA	Choice experiment (online survey) (‘Please choose the option that you prefer’; displaying the same four-pack yoghurt with different labels)	US adult consumers	Sustainability: USDA organic logo; Carbon trust label. Nutrition: ‘Health claim’	Yoghurt (made of cow’s milk)	Time preferences (delayed gratification and related concepts); Preference for foods carrying various nutrition and/or sustainability labels.	Consumers derive the higher utility from the presence of the USDA organic logo, followed by the presence of the disease risk reduction claim, the carbon trust label and last from low energy contents. Individuals with low time preferences (higher orientation toward the future) are more careful about the organic logo and low energy contents, whereas individuals with high time preferences are not.
de-Magistris, T. *et al* (2016)^(37)^	Spain	Non-hypothetical choice experiment	Spanish adult consumers	Sustainability: EU organic logo; – ‘Distance claims’	Almonds	Consumers’ preferences and willingness to pay	Spanish consumers are Willingness to Pay a (statistically significant) price premium for almonds labelled either EU organic, or 100 km distance claim. They were not Willingness to Pay extra for the 800 km claim, and the 2000 km claim had a negative impact on their will to buy the product.
Delpozo, B., *et al* (2025)^(43)^	Spain	Eye-tracking experiment combined with blind *v*. informed taste tests	General consumers	EU-O logo and a novel ‘SI’ logo	EVOO	Liking expectations, actual liking, purchase intention, visual attention	Both logos created a positive ‘halo effect’, increasing expected and actual liking. Consumers paid more attention to the familiar EU-O logo, which was linked to improved expectations.
Duckworth, J., *et al* (2022)^(44)^	United Kingdom	Online experiment (choice experiment, contingent valuation)	1071 meat-eating UK consumers across two experiments	Hypothetical text-based ‘green labels’ (e.g. ‘Sustainably sourced’, ‘Locally sourced’)	Various foods (meat, fish, dairy, vegetables, fruit)	Food choice, Willingness to pay (WTP)	Green labels’ massively increased choice (344 %) compared to no label and modestly increased WTP. ‘Sustainably sourced’ and ‘Locally sourced’ were the most preferred labels.
Fernández-Serrano, P., *et al* (2022)^(45)^	Spain	Online questionnaire/choice task; eye-movement tracking.	Regular wine consumers	Sustainability: Sustainably irrigated label (FOP and BOP alternatives; logo *v*. text alternatives) Labels were specifically designed for this study	Wine	Willingness to pay (for a sustainability labelled wine bottle)	The choice task results showed that almost 90 % of the participants were interested in the labelled wines. They preferred the logo version (both on FOP situation or BOP situation). On average, they were Willingness to Pay more for wines with the label. Eye tracking revealed an interest in the label, and it was positively correlated with WTP (price premium) for the labelled wine.
Fretes, G., *et al* (2021)^(57)^	Chile	Activity-based online focus group discussions	Children (average age 8·3 years in the study)	Sustainability: Three made up levels showing various label designs: ‘Yo cuido al planeta’ [I care for the planet]; ‘Bueno para el planeta’ [Good for the planet]; ‘Alto - medio - o bajo impacto’ [High – medium – low impact]; Other labels displaying carbon footprint	Multiple food items, including fruits, flavoured yoghurts, processed snacks and packed fruit juice	Willingness to pay (to buy); Awareness of sustainability labels or concepts	Taste was the main driver of children’s favourite snacks. Yet, a simple eco-label in the form of a sticker including elements typically associated with the environment (such as the planet, hands or leaves) and a short message was well understood by children. Most children could differentiate which product was ‘better for the planet’. Amongst children who usually receive pocket money, WTP generally matched the actual market price of the product.
Hallstein, E. *et al* (2013)^(51)^	USA	Experimental pre- and post-‘treatment’ design (collection of point-of-sales data from a various supermarkets)	Adult consumers (general population)	Sustainability: ‘FishWise Advisory’ label (traffic light system with seafood production or capture method depicted)	Seafood	Real-life purchasing decisions (sales measured by weight and $)	The advisory led to a statistically significant 15·3 % decline in overall seafood sales, a statistically significant 34·9 % decline in the sale of yellow labelled seafood, and a statistically significant 41·3 % decline in the sale of yellow labelled seafood on a mercury safe list. No statistically significant difference in sales of green or red labelled seafood was found.
Johnston, R. J., *et al* (2001)^(56)^	US and Norway	Telephone survey	Adult consumers (main shoppers in seafood consuming households)	Sustainability: ‘Eco-label’ certified (text saying: ‘caught under strict controls that prevent too much fishing’). (Endorsed by the World Wildlife Fund, the Marine Stewardship Council and the National Marine Fisheries Service.	Cod and shrimp (seafood)	Willingness to pay (a price premium)	US consumers were Willingness to Pay for a higher price premium compared to the Norwegian consumers, but both groups assigned more value to the certified products.
Jürkenbeck, K., *et al* (2023)^(46)^	Germany	Online survey with choice experiments	German consumers	Nutri-Score, Eco-Score, nutrition claims and organic labels	Strawberry yoghurt and oat crunchy muesli	Consumer preference, Willingness-to-pay (WTP)	Consumers showed high preference for interpretative labels like Eco-Score and Nutri-Score. WTP was highest for ‘A’ rated labels, while ‘C’ rated labels had no effect compared to no label.
Kaczorowska, J., *et al.* (2019)^(47)^	Poland	Online survey (behavioural choice experiment)	Adult urban consumers	Sustainability: EU organic farming (Euro leaf); Protected geographical indication; Fair Trade (Each *v*. a control without a sustainability logo).	Rice, beans, apples, bananas	WTB; Willingness to pay (WTP)	When the logo was poorly known, even consumers with positive attitudes toward sustainability did not use it as a cue when shopping for food. Urban consumers were very price sensitive and showed a restrained desire to pay a higher price for sustainability labelled products. One-third of them had a higher WTB and WTP for such labelled food.
Lamonaca, E., *et al* (2022)^(28)^	Italy	Online survey	Adult Italian consumers	Sustainability: Organic label Environmental label Quality label Nutrition: Health claim label	N/A	Perceived quality, healthfulness and safety of organic food	Consumers perceived safety of organic food better than healthiness and environmentally sustainable attributes. The more details on the labels the higher likelihood of perceiving organic food as healthier, safe and environmentally sustainable. Younger consumers were more prone to buy and consume organic products.
Latip, M., *et al* (2024)^(58)^	Malaysia	Cross-sectional correlational study (online survey)	Malaysian consumers	Organic food labelling (in general)	Organic food (in general)	Purchase intention, personal attitude, satisfaction with labelling	Personal attitude strongly influences purchase intention. Satisfaction with existing labels has a positive impact, while a desire for more labelling information has a negative impact on attitude and intention.
Lazzarini, G. A., *et al* (2016)^(29)^	Switzerland	Experimental design (In-person food sorting task where participants categorised food products according to their healthfulness and environmental friendliness).	Swiss adult consumers	Sustainability: Organic label	Protein-rich foods (mainly animal and some plant-based)	Perceived healthfulness. Perceived environmental friendliness	The presence of an organic label was an important criterion for environmental friendliness. The presence of an organic label also played a significant role for healthiness estimations. Origin was another important criterion of environmental friendliness among the participants.
Maier, M. (2024)^(48)^	Switzerland	Survey experiment	Swiss residents	Information about a retailer’s existing CO2 food label (’Mcheck’)	General food consumption (intentions)	Impact on the uptake of plant-based diets, food consumption behaviour and social acceptance	Providing information about the voluntary label increased consumers’ intention to reduce their carbon footprint and significantly increased support for a mandatory governmental CO2 food label.
Marette, S. (2021)^(30)^	France	Online survey	French adult population	Nutrition: Nutri-Score; Sustainability: Eco-score; ‘Global-score’ (aggregate health + sustainability score).	Pizza	Purchasing intentions	For each type of label, the dominant effect comes from the reduction in purchase intentions related to the red colour, with Nutri-Score showing the strongest effect. Green or yellow colours also changed purchase intentions but to a lesser extent. Only 53 % of the sample thinks that Global score summarising the health and eco-labels would be relevant.
Nguyen, M., *et al* (2018)^(59)^	Vietnam	Experimental auctions	Vietnamese adult consumers	Sustainability: VietGAP label (Three alternatives: on its own, plus supplementary information or plus supplementary and traceability information).	Rice	Willingness to pay	Domestic consumers were Willingness to Pay a 9 % price premium for certified sustainably produced rice. This premium gradually increased up to 33 % when incremental levels of information on certification and traceability were provided.
Paffarini, C., *et al* (2025)^(63)^	Italy, Greece, Israel	Discrete Choice Experiment via online survey	Regular consumers of EVOO	Protected designation of origin, organic and carbon footprint labels, alone and in combination	EVOO	Consumer preference, Willingness-to-pay (WTP), multi-label interaction effects	A combination of two labels sometimes had a synergistic (superadditive) effect on WTP, but combining three labels had a diminishing (sub-additive) effect, suggesting information overload.
Pink, A. E., *et al* (2022)^(52)^	United States	Online choice experiment	Adult (> 21 years/old) consumers from the US	Nutrition: HENI label; Sustainability: Carbon footprint score.	Multiple food products	Purchasing intentions. Willingness to buy (WTP – ‘intentions to change’)	Intentions to increase consumption of foods that were beneficial to health, but high risk for the environment was highest for participants provided with only HENI information. Participants presented with carbon footprint information only reported the greatest intentions to decrease consumption of such foods. Intentions to change scores reported by the environment condition were significantly lower than all other conditions, which suggests participants prioritise the health benefits of foods over the environmental sustainability of them.
Risius, A., *et al* (2017)^(31)^	Germany	A combination of qualitative research methods, with think-aloud protocols and in-depth interviews, as well as quantitative methods, using choice experiments and face-to-face interviews	Adult regular consumers of fish who do not work in the industry	Sustainability: EU organic label; German governmental organic label; Organic farmers’ association label; Naturland; The label of the Aquaculture Stewardship Council; World Wide Fund For Nature; ‘Company owned’ label.	Fish (smoked trout fillets)	Consumers’ preferences. Consumer’s expectation of sustainability labelled aquaculture products	Consumers don’t use labels as decisive elements when purchasing aquaculture products. ‘Geographical origin’, ‘price’ and ‘health claim’ were more important to consumers than ‘sustainability’ labels. Sustainability of aquaculture was associated with natural, traditional, local and small-scale production systems with high animal welfare standards.
Scozzafava, G., *et al* (2020)^(32)^	Italy	Visual discrete choice experiment (online apparently)	Italian adult consumers	Sustainability: Organic label (not the EU nor an ‘official’ label, with 4 different variants of additional text information)	Cow’s milk	Consumer preferences; Willingness to pay (WTP)	Treatments on quality and production costs had no effect on consumer preferences for organic milk. Consumers who received information on animal welfare or about environmental sustainability in organic farming showed a greater WTP for organic milk than for conventional milk. When no additional information was provided (product only displayed ‘organic’ text), there was no preference for organic milk over conventional milk.
Shaik, S., *et al* (2024)^(54)^	United States	Two controlled online experiments	Adults	All-encompassing label (Eco-score B) *v*. a one-trait label (organic)	Cereal and bread	Willingness-to-pay (WTP); Perceived self-benefits	The organic label resulted in higher WTP than the Eco-score B because it signals direct self-benefits (perceived healthiness), which the more abstract Eco-score does not.
Sun, X., *et al.* (2024)^(62)^	China	Real-world field experiment in university canteens	University students and faculty	Colour-based carbon footprint labels and energy content labels	Dumplings	Sales data (purchasing behaviour)	Environmental (carbon) labelling significantly reduced purchases of high-carbon dumplings. Women were more influenced by environmental labels, whereas men were more sensitive to energy labels.
Taillie, L. S., *et al* (2021)^(53)^	United States	Randomised online survey	USA adult red meat consumers	Sustainability: Environmental warning; Nutrition: Health warning; And a combined health and environmental warning (*v*. a no-label control)	Burritos (red meat, chicken, and vegetarian)	Food preference; Capacity to discriminate the least healthy item; Capacity to discriminate the least environmentally friendly item	There were no significant differences in selection of the steak burrito between label conditions or in selection of the item most damaging to the environment. Those exposed to the health warning were more likely to select the steak burrito as most damaging to health compared to those exposed to other label conditions. The combined and health warnings elicited higher perceived message effectiveness ratings than the environment warning. Warnings did not have a significant effect on item preference in the choice experiment.
Taillie, L. S., *et al* (2024)^(55)^	United States	Online randomised experiment	USA adults	Eco-score label (colour-coded, A-F grade) *v*. a barcode control	Sandwiches, pizzas, snacks and burgers	Perceived environmental sustainability; Purchase intentions	Eco-score labels significantly increased perceived sustainability for sustainable products (+16 %) and decreased it for unsustainable products (−13 %). The effect on purchase intentions was smaller but still significant.
Tait, P., *et al* (2016)^(61)^	UK and Japan	Choice experiment (assessing stated preference)	Japanese and British adult consumers	Sustainability: Text-only label; Text + graphics label; Sustainability compass label; These were developed for this study specifically, with the intention to represent the core characteristics of eco-labels.	Fruit products	Willingness to pay	Environmental sustainability attributes were important contributors to consumers’ fruit choices, irrespective of the label format used. Carbon labelling was one of the most important attributes for both countries’ consumers across all formats. The influence of presentation format was most apparent between text-only and compass formats. While WTP for carbon was found to be insensitive to format, the reverse result is found for WTP for vitamins.
Van der Waal, N. *et al* (2022)^(33)^	Netherlands	Pilot study involving a one-factor experimental design. (Shopping task using an online supermarket)	Adult Dutch consumers	Sustainability: Sustainability (text-only) claim; Explanatory (text-only) sustainability claim; Explanatory sustainability claim + health claim	Multiple: bread, dairy and sandwich filling, pasta, vegetables, meat and vegetarian alternatives and sauces	Purchasing intentions	The addition of a health claim to a sustainability claim on food products did not result in more sustainable purchases. An explanatory sustainability claim did not increase sustainable purchase behaviour among either consumers with high or low environmental attitudes. Adding explanatory sustainability information did more harm than good for consumers’ sustainability purchases with low environmental attitudes.
Vlaeminck, P., *et al* (2014)^(34)^	Belgium	Online survey (pretest six potential labels) + a field experiment (using the best performing label)	Adult consumers	Sustainability: Eco-label (developed for the study)	Multiple: fruits (apples), vegetables (tomatoes), and ‘protein’ (steak, chicken and veggie burger)	Purchasing behaviour	The new label that was preselected in the online survey leads to more eco-friendly food purchases, relative to either the label currently used in the supermarket or the label that contains the raw information of the environmental impact. In the experimental food market, the use of an easy-to-interpret environmental information label increases the overall eco-friendliness of the subjects’ food choices by about 5·3 % relative to the default label currently used.
Weinrich, R., *et al* (2016)^(35)^	Germany	Online surveys (two subsequent experiments)	Household decision makers	Sustainability: Animal welfare label of the German Animal Protection Association	Pork (fresh meat and processed meat)	Willingness to pay	WTP was significantly higher for premium products with two stars than for products labelled with one star. When an explanation about the levels of the label is provided, there is an increasing willingness to pay for products with higher standards of animal welfare. The study also supports the notion that a multilevel label enhances information overload, and a multilevel label is not comprehensible unless additional information is provided.
Weinrich, R.; *et al* (2016)^(36)^	Germany	Survey (five variables in relation to use of food labels and a comparison between a binary and a multilevel label, against a non-labelled products)	German adult consumers	Sustainability: Animal welfare	Meat (beef)	Consumers’ satisfaction; Intention to buy (measured indirectly through the survey questions)	There were no statistical differences between the two labelling schemes for the latent variables comprehensibility, involvement and use. There were differences for time pressure and trust. The group comparison indicated that trust as a precondition is more necessary for a binary label whereas high time pressure reduces satisfaction with a multilevel label.
Williams, V., *et al* (2023)^(22)^	United Kingdom	Mixed-methods (online questionnaire and semi-structured interviews)	UK adults	Eco-score label (‘Eco-Impact’ version by Foundation Earth)	Meat products	Label perceptions, purchase intentions, drivers and barriers	Meat attachment (affinity for meat) had a negative relationship with purchase intentions for eco-labelled meat. Key barriers to using the label were habitual shopping behaviours and the perceived higher price of sustainable options.

EF, Environmental Footprint; ASC, Aquaculture Stewardship Council; RCT, Randomised controlled trial; EU-O, EU-organic; EVOO, Extra virgin olive oil; SI, sustainable irrigation; FOP, Front-of-pack;, BOP, back-of-pack; WTB, Willingness to buy; HENI, Health Nutrition Index.

The critical appraisal tool showed that most studies had clear aims and were grounded on sound theoretical concepts. In general, study designs adequately addressed the stated aims but information on sampling was lacking enough detail in several studies. Data collection and data analysis procedures were appropriate for most studies and were usually described in depth. Very few studies had any form of stakeholder engagement; the vast majority did not provide any details on this. Although most studies critically discussed strengths and limitations, some were rather general, and a few did not mention them at all. For detailed information, see Table A2 in the appendix.

### Labels’ features

Studies assessed labels currently available in the market (*n* 20)^(23,24,27,30,31,35–37,39,40,43,46,47,49,50,54,55,59,60,62)^, and others analysed tailored or hypothetical labels (*n* 21) exclusively designed depending on the research aims^(22,25,26,28,29,32–34,38,41,42,44,45,48,51–53,56–58,61)^. The existing labelling schemes for environmental sustainability and social responsibility assessed were as follows: *Eco-Score* (*n* 7)^(27,30,39,40,46,54,55)^, *EU organic* (*n* 5)^(23,31,37,43,47)^, Aquaculture Stewardship Council *logo* (*n* 2)^(24,31)^, *Carbon Trust Label* (*n* 2)^(23,50)^, *EU agriculture* (*n* 2)^(23,24)^, *USDA organic* (*n* 2)^(49,50)^, *Fairtrade* (*n* 1)^(47)^, *World Wildlife Fund* (*n* 1)^(31)^, some labels specific to certain countries, such as the *Colorado Proud*, *rBST-free* and *Validus* labels^(49)^, and the German organic label, such as *Organic Farmers’ Association* and *Naturland* ^(31)^. Some studies also included well-known nutrition/health labels, such as the *Traffic light* (for nutrients) (*n* 3)^(23,25,41)^ and *Nutri-Score* (*n* 3)^(27,30,46)^, while some used various types of health claims (*n* 8)^(24,28,30,31,33,50,52,56)^ and/or environmental sustainability claims (*n* 6)^(22,44,48,56,58,60)^ for their research. The features of existing labelling and most assessed schemes are presented in Table 3.

**Table 3.** Main features of the existing and most assessed labelling schemes in terms of nutritional, environmental sustainability and social responsibility domains

Label	Primary focus	Key features
Aquaculture Stewardship Council logo	Responsible aquaculture (farmed seafood)	Text-only. Certifies that farmed seafood meets standards for environmental responsibility (e.g. water quality and biodiversity). Guarantees social responsibility, including fair treatment of workers and community engagement.
Carbon Trust Label	Carbon footprint (Greenhouse gas emissions)	Logo with human foot. Sometimes includes CO2 g per unit of product. Certifies that a product’s carbon footprint has been measured to a recognised international standard
Eco-score	Overall environmental impact	Front-of-pack label featuring a colour-coded, letter-graded scale from A (dark green) to E (dark red). The design is intentionally similar to the Nutri-Score to leverage consumer familiarity. The logo typically includes a leaf symbol within the letter grade, alongside the full A-E colour spectrum to provide context
EU agriculture	Geographical origin	Mandatory text indicator that accompanies the EU Organic logo. Specifies if agricultural ingredients are from within the EU, outside the EU or a mix of both.
EU organic	Organic farming standards	Commonly known as the ‘EU green leaf’. It features the stars of the European Union arranged in the shape of a leaf against a green rectangular background. Its use is compulsory for all pre-packaged organic foods produced in the EU
Fairtrade	Social & Economic equity	Green, blue and black stylised figure representing a person, with ‘Fairtrade’ text. Guarantees a Fairtrade Minimum Price to protect producers from market volatility. Provides an additional Fairtrade Premium for producers to invest in their communities Ensures decent working conditions and bans child and forced labour.
Nutri-Score	Nutritional level	Front-of-pack label that provides a single, overall rating of a product’s nutritional quality on a five-level scale, using both colours (from dark green to dark red) and letters (from A to E).
Traffic Light	Nutritional level	Front-of-pack system that uses the intuitive colours of traffic lights (red, amber and green) to provide information on individual key nutrients, such as fat, saturated fat, sugars and salt. It does not provide a single summary score but rather a colour-coded rating for each nutrient.
USDA organic	Organic farming standards (USA)	Official organic seal of the United States Department of Agriculture Prohibits synthetic fertilisers, pesticides and GMO. The seal is circular and features a green outer ring and a brown inner ring, with the words ‘USDA ORGANIC’ written in white. Certifies strict standards for soil quality and animal raising practices.
World Wildlife Fund	Conservation support	Panda symbol. It represents corporate partnership, not a formal product certification.

GMO, Genetically Modified Organism; EU, European Union; USDA, United States Department of Agriculture.

Regarding label features and their impacts, traffic light-style warning labels on food products have shown effectiveness, particularly with the red signal being especially persuasive in discouraging undesirable attributes of food items. The red colour heightened respondents’ inclination to avoid the most unfavourable levels of collective attributes such as climate impact or antibiotics use, potentially linked to loss aversion and negativity bias in consumer psychology, while the green colour strengthened preferences for healthiness^(25,46,56)^. In Spain, a study found that carbon footprint labels garnered more attention when perceived as a hazard cue, especially when displayed in a red warning sign^(26)^. Despite the success of warning labels in adequately signalling GHG attributes to consumers, the overall effect of those labels on purchasing intentions is mixed. Compared to other labels, one study showed that consumers derived a higher utility from the organic label and health labels/claims, ranking the *Carbon Trust’s* carbon footprint label in third place^(50)^. Another study found that the impact of this carbon footprint label is secondary to price and fat labels on meat products^(23)^. Yet, in two studies, carbon labelling had a strong positive influence on respondents’ WTP for various food products^(52,61)^.

### Labels’ impacts

In general, studies showed that environmental sustainability labels tend to have a positive outcome, eliciting a higher WTP, stated preference or product choice^(25,36,37,41,44,48–52,56,59,61)^. However, their effectiveness can vary by country, with some consumers in Italy showing less attention or willingness to pay a premium for carbon footprint certification compared to well-known labels like organic or protected designation of origin^(60)^; consumers in Germany were not decisive for fish purchases^(31)^, and a study in the Netherlands where an explanatory claim resulted in less sustainable food purchases for consumers with low environmental attitudes^(33)^.

‘Organic’ labels were consistently valued or preferred by consumers^(28,29,43,50,58)^. However, consumers often ascribed qualities to these labels that they do not necessarily have. For example, products labelled organic were thought of as more environmentally friendly^(29)^, healthier or safer^(28)^, a phenomenon known as the ‘health halo effect’^(65)^. One study assessing WTP found that organic labels were valued more highly than specific environmental sustainability labels, such as CO_2_ labels^(37)^.

Consumers usually prefer additional information about the certification criteria and traceability of foods. A study in Vietnam that assessed the WTP of rice labelled with a local government-endorsed environmental sustainability label found that consumers were Willingness to Pay a 9 % price premium for certified rice, and this figure increased up to 33 % when incremental levels of information about the label were provided^(59)^. Similarly, a study in Italy assessing preference for organic labelled *v*. conventional cow’s milk found that preference for the former was significant only when additional information about environmental sustainability was provided^(32)^. Similar results were seen in a study that evaluated WTP for foods with an animal welfare label in Germany^(35)^. Conversely, other studies highlight the potential for information overload or confusion when multiple, comprehensive labels are present. One study found that all-encompassing sustainable labels were not always the most effective, suggesting that detailed information about energy cost, carbon emissions and other attributes could overwhelm consumers, diminishing interpretation and influence on WTP^(54)^. Another study also reported a significant negative interaction (sub-additive effect) when three labels were combined^(60)^.

Another important finding is that participants seem to prioritise the health benefits of food over its environmental sustainability attributes. This was reported in a study evaluating various types of meat products in the UK, where price and fat content labels had a larger impact on consumers’ choices than environmental sustainability labels^(23)^, and in a study in the USA, where participants reported much lower intentions to change their food choices when presented a carbon footprint score *v*. a health nutrition index^(52)^.

Studies suggest a discrepancy between what people say they value and what they choose or buy. A systematic review assessing consumers’ preferences found that environmental and social responsibility attributes were ascribed a higher utility or rank to nutrition attributes in 17 studies, compared to nine where nutrition attributes were valued more highly^(66)^. This contrasts with the findings that displayed the *Nutri-Score* and *Eco-Score* labels at product level, which led to improved nutritional quality of food choices but did not reduce the environmental impact of these, despite the respondents reported having strongly relied on both scores for their choices^(67)^. It also contrasts with a study in France, where purchasing intentions for pizza were significantly more sensitive to the *Nutri-Score* label than for the *Eco-Score* or a combination of the two called ‘*Global Score*’^(30)^.

Animal welfare emerged as a very important topic for consumers. One study found that the price premiums for a green label compared with a red label were 52 % for healthiness, 64 % for both animal welfare and antibiotics and 20 % for climate impact^(25)^. Another study in the USA assessing WTP for cow’s milk under various environmental sustainability labelling schemes showed that nearly all participants placed animal welfare at the top of their priority list^(49)^. One study in Germany exclusively assessed WTP for an animal welfare label and found that consumers were Willingness to Pay more for pork products displaying this label, especially when an explanation of the label was provided^(35)^.

Real-life purchasing decisions seem to be scarcely evaluated. One study in Belgium evaluated the performance of an environmental sustainability ‘eco-label’ specially developed for the experiment. The label was preselected by participants of an online survey and then tested by looking at purchasing behaviour among consumers in a supermarket. Although the label was tested in a controlled environment under predefined experimental conditions, it increased the overall ‘eco-friendliness’ of the subjects’ food choices by about 5·3 % relative to the default label currently used in the market^(34)^. Another study assessing real-life purchases was conducted in the USA and measured the impact of a ‘Fish Wise Advisory’, a traffic light label depicting the capture or production methods of seafood. The advisory led to a statistically significant 15·3 % decline in overall seafood sales and a statistically significant 34·9 % decline in the sale of yellow labelled seafood, but no significant difference in sales of green or red labelled seafood was found^(51)^. Despite these real-life purchasing studies, new studies increasingly utilised more realistic settings, such as virtual reality supermarkets^(38)^ and university canteens^(56)^, reinforcing findings on actual behaviour rather than just stated preferences.

## Discussion

To the best of our knowledge, this is the first systematic review that identified the features and impacts of environmental sustainability and social responsibility food labels on consumers’ choices and purchasing decisions. The growing body of literature, particularly from 2022 to 2025, underscores the increasing global attention to these labelling schemes as tools for promoting sustainable diets. Also, the increasing number of studies from diverse geographical regions provides valuable context and suggests that the fundamental principles of label effectiveness may transcend specific cultural contexts, though specific label preferences and willingness to pay can vary significantly by country. However, there remain several challenges to comprehensively understand the effects of the impacts of environmental sustainability and social responsibility on food labels, especially due to the wide range of methodological approaches, study designs, and sampling and populations.

Overall, demographic factors such as age, gender and socioeconomic status can play a role in how consumers perceive, understand and respond to environmental sustainability and social responsibility food labels, though the specific effects can vary depending on the label type, product, and study context. Most of the included studies assessing gender differences show that women have higher WTP for foods with environmental sustainability labels than men. This aligns with the findings of a recent meta-analysis looking at WTP for short food supply chain products^(68)^. We did not find a clear pattern by age group; however, a review focusing on discrete choice experiments found that people up to 40 years old were more responsive to these labels compared to older individuals^(69)^.

While consumers express positive attitudes and WTP for environmental sustainability labels, this sentiment does not consistently translate into real-life purchasing decisions or behaviour change. This discrepancy is recognised by environmental psychology and human behaviour research as a ‘value-action gap’ in environmental sustainability, where people’s stated concerns do not always align with their actions^(70)^. Some studies revealed a higher preference for environmental sustainability and social responsibility labels over nutrition labels^(41,50)^, while others indicated a prioritisation of nutritional quality over environmental sustainability attributes^(30,52)^, despite an expressed value for the latter^(67)^. Evolutionary perspectives on cognition argue that this happens because humans apply heuristics, originally shaped to handle social exchange, on environmental impact issues^(71)^. Consequently, a misconception arises whereby sustainable choices can offset unsustainable ones, potentially influencing attitudes toward food labels. Additionally, social desirability bias, though minor in environmental psychology^(72)^, may play a substantial role in surveys regarding sustainable food consumption^(73)^.

A consistent preference for foods displaying an organic label is observed, which could be an encouraging sign for global environmental sustainability, but has important nuances. Confusion exists surrounding the term ‘organic’ in labels, as these products are often ascribed a health halo by consumers^(40,43,54,65)^ despite limited evidence supporting higher health benefits^(74)^. Moreover, there is no consensus whether organic truly means environmentally sustainable, considering that the same output of organic food requires more land than conventional agriculture (although it reduces fertiliser surplus and pesticide use)^(75)^. From the perspective of the Sustainable Development Goals, organic farming relates to several goals; however, the question remains as to whether a transition to a predominantly organic global food system is achievable and will it be able to feed a growing world population.

GHG or carbon footprint labels received little, but increasing attention in studies across this review, highlighting the potential mismatch between individual and global priorities, such as curbing carbon emissions to address climate change. Information related to GHG emissions and its relevance needs to be presented in a manner that is more effective to consumers to include this dimension in their choices.

Consumers place great importance on place of origin of food products^(24,29,31,44)^. From the standpoint of GHG emissions, this is not the most important dimension, as research shows that, on average, between 1 % and 7 % of the GHG emissions of a given product derive from transportation^(76)^. However, indicating the place of origin on a label could still have other relevant values, such as increasing the resilience of local production systems, and potentially, the preservation of biodiversity and less water use. These need to be explained in the labelling scheme, as consumers probably associate local products with traditional, ‘natural’ or small-scale low-impact production systems, even when this might not be the case.

According to this review, animal welfare is a top concern for consumers^(25,32,35,49)^, but not for global leaders. The Sustainable Development Goals fail to mention this dimension of environmental sustainability; however, its importance was recently recognised by the United Nations. Since people assign a high value to animal welfare, including this dimension in an integrated FOP food labelling system seems an important endeavour. Also, it is notable how prominent animal welfare is in this review and how issues of human welfare or social responsibility are barely considered. Studies assessing social responsibility dimensions were few and their findings were inconsistent across this review^(47,49)^, showing that more research is needed.

Achieving healthier, more environmentally sustainable, and socially responsible food choices through food labels often implies consumers changing their behaviours or preferences. Even though it is recognised that human behaviour change is complex and challenging, public policy often operates based on an information-deficit model, whereby it is assumed that if people are informed of the benefits or harms of certain food products, they will change their behaviours accordingly^(77)^. However, information alone and/or abundance of information is unlikely to change consumers’ eating habits^(78)^ and actually can be even more detrimental for facilitating choices^(39,42,58,60)^.

A set of diverse approaches are needed to inform people about the food choices in terms of health, environmental sustainability and social responsibility concerns. Public policies need to consider well-planned and designed food labels along with other public measures, such as taxing sugar-sweetened beverages to improve health^(79)^, addressing overfishing by diverting subsidies from industrial fisheries^(80)^ or reducing the marketing of unhealthy foods to children^(53)^, Additionally, factors like price and social norms influence food choices and should be taken into account when designing effective policies.

There is a growing need for improved food labelling that addresses the various impacts of food systems^(81)^. However, studies suggest that using multiple nutrition or environmental sustainability labels on a product might lead to confusion and potentially negatively affect consumer preferences^(39,42,58,60,82)^. Given these challenges, the development of a single integrated food label that considers nutrition, environmental sustainability and social responsibility could be a valuable tool for public health. Recent research shows that there is widespread public support for a universal ‘eco-label’ to be introduced to support better food choices, which also may address *greenwashing* concerns and issues of trust in governments current regulatory standards^(83)^.

However, despite policymakers and stakeholders in the European Union advocating for a harmonised FOP system (i.e. *Farm-to-Fork* strategy), they have had difficulties in reaching an agreement^(84)^. Although diverse food labels show similar results across countries, there is also an important gap in information regarding how FOP labels work across different population groups and cultures, particularly in low- and middle-income countries where evidence is more scarce and barriers related to governance and literacy may hinder the implementation of these labels^(85)^.

Overall, this review has shown that evidence on this topic is diverse, limited, geographically uneven, but growing. In this sense, several areas for future research and development can be drawn. First, a significant limitation in existing research is the lack of studies measuring real-life evaluations. Hypothetical choice experiments, while valuable, may not fully capture the complexity of real market situations. Therefore, there is a need for moving beyond stated preferences to assess real-life evaluations. Second, the sub-additive effects observed with multiple labels suggest that simply adding more information or certifications does not always lead to better outcomes, potentially confusing consumers. In this way, there is a need for addressing the impact of information overload and label combinations. Complementarily, the efficacy of labels is influenced by their design features, including whether they are interpretative or descriptive, nutrient-specific or summary scores, and use warning or positive indicators. Developing integrated food labels that encompass nutrition, environmental sustainability and social responsibility dimensions requires a series of potential elements for this integrated profiling system. Based on this review and previous research^(10,19,27)^, Table 4 suggests, but not limits, the main the potential elements that could be included in such a profiling system.

**Table 4.** Potential elements to include in the profiling system of an integrated planetary health food label encompassing nutrition, environmental sustainability and social responsibility dimensions

Dimension of the label	Element of the profiling system
Nutrition	Energies per serving
	Total fat: saturated fat and trans fat
	Cholesterol
	Total carbohydrates: dietary fibre, total sugars and added sugars
	Protein
	*n*-3
	Key micronutrients: iron, folate, calcium, vitamins (B_12_, D, A and C)
	Degree of food processing (minimally processed – ultra-processed)
Environmental sustainability	CO_2_ emissions associated (as CO_2_ equivalents)
	Water use
	Distance travelled
	Associated land use
	Waste generated
	Pesticide intensity
	Fertilisers intensity
Social responsibility	Degree of animal welfare
	Local development index
	No exploitation index
	Fair payment index
	Community resilience index
	Degree of food security of producers

### Limitations

This study has several limitations. The identified labels were not classified as endorsed by a government or by a private organisation, which is important since this relates to issues of trust and transparency, which have shown to be essential for labels to be truly impactful on consumers’ behaviours^(81)^. Additionally, nutrition/health labelling studies were not actively searched for in this work because various systematic reviews have already been conducted and there is notoriously more evidence regarding this dimension^(10,19,27)^. But still, valuable information may have been retrieved by including this element. Also, much of the literature about food labelling may be found in policy documents, government reports and other types of files from the ‘grey’ literature. This was explored in this work to inform the background and discussion, but a more systematic approach could have been employed.

Another limitation is related to the sample sizes and sampling techniques among the included studies. These samples ranged from ∼100 to ∼3000 participants, including random to purposive samples with unbalanced participants’ characteristics. This heterogeneity might affect the overall effects and should be considered in future studies. Additionally, the lack of articles that measured real-life evaluations is considered as a limitation. Overall, research should move beyond consumers’ stated preferences, to assess real-life purchasing behaviours and employ methods that more closely resemble how consumers interact with labels. Given the geographical distribution of articles, more studies should be conducted in low- and middle-income countries to identify what are the top concerns in those contexts and whether there is some universality as to how labels operate across the world. To build an integrated food label, a transdisciplinary approach is needed, which includes researchers, policymakers and users to find out what is the best way to proceed and achieve a labelling system that truly serves its purpose.

### Conclusion

There is limited and mixed evidence evaluating the impacts of environmental sustainability and social responsibility on food labels. This systematic review highlights the complex and often inconsistent relationship between consumers’ attitudes toward environmental sustainability and social responsibility food labels and their actual purchasing behaviours. While environmental sustainability labels generally lead to positive outcomes, such as increased willingness to pay (WTP) and preference for labelled products, a significant ‘value-action gap’ persists. This gap, where consumers’ stated values do not align with their real-life choices, emphasises the need for a deeper understanding of the cognitive and psychological factors influencing food decisions. The strong preference for organic and animal welfare labels, despite potential misconceptions about their true environmental and health benefits, underscores the potential for ‘health halo’ effects to shape consumer perceptions.

The review also discusses the potential elements for an integrated ‘planetary health food label’, which is food labelling system that encompasses nutrition, environmental sustainability and social responsibility. Such a system could provide consumers with clear, consistent information and could help mitigate confusion caused by multiple labels. However, achieving consensus on a universal label remains challenging, and diverse approaches are required, including public measures and the influence of social norms, to foster healthier, more sustainable food systems.

## Supporting information

Izcue and Palmeiro-Silva supplementary material Izcue and Palmeiro-Silva supplementary material

## Supplementary material

For supplementary material accompanying this paper visit https://doi.org/10.1017/S1368980026101979
