# Supplementary material for: A systematic review on how environmental sustainability and social responsibility food labels influence consumers’ food choices and purchasing decisions
Source: Public Health Nutr. 2026 Feb 11;29(1):e62. doi: 10.1017/S1368980026101979 (PMC13087989; doi:10.1017/S1368980026101979)
Supplement: Izcue and Palmeiro-Silva supplementary material [file S1368980026101979sup001.docx]

**Suplementary Material - Appendix**

**A Systematic Review on How Environmental Sustainability and Social Responsibility Food Labels Influence Consumers’ Food Choices and Purchasing Decisions**

[Insert authors’ names here after peer-review]

**Table A1.** Keywords and full search query (with truncation) used across the databases

| **Food labels** | **Impact** | **Sustainability** | **Social responsibility** |
| --- | --- | --- | --- |
| - Food labels - Nutrient profiling system - Food score - Front of pack nutrition label - Food metric | - Impact - Effectiveness - Influence | - Sustainable - Land-use change - Water use - Greenhouse has - Environmental footprint - Ecological footprint - Food footprint | - Social justice - Social responsibility - Fairness - Fairtrade - Equity - Livelihood |

**Table A2.** Critical appraisal of articles included in the systematic review according to QuADS guidelines.

| **First author** | **1 Theory** | **2 Aim** | **3 Research setting** | **4 Study design** | **5 Sampling** | **6 Data collection (rationale)** | **7 Data collection (format)** | **8 Data collection (procedure)** | **9 Recruitment** | **10 Analytic methods** | **11 Method appropriate** | **12 Stakeholder** | **13 Strengths and limitations** |
| --- | --- | --- | --- | --- | --- | --- | --- | --- | --- | --- | --- | --- | --- |
| Apostolidis, C. et al. (2016)^1^ | 3 | 3 | 3 | 3 | 1 | 3 | 3 | 3 | 2 | 3 | 3 | 0 | 3 |
| Arrazat et al. (2023)^2^ | 3 | 3 | 3 | 3 | 3 | 3 | 3 | 3 | 3 | 3 | 3 | 1 | 3 |
| Banovic, M., et al. (2019)^3^ | 3 | 2 | 3 | 3 | 1 | 3 | 3 | 3 | 1 | 3 | 3 | 0 | 3 |
| Berden & Hung (2025)^4^ | 2 | 3 | 2 | 2 | 2 | 2 | 3 | 3 | 2 | 3 | 3 | 0 | 2 |
| Büttner, V., et al (2024)^5^ | 3 | 3 | 2 | 2 | 3 | 2 | 3 | 3 | 3 | 3 | 3 | 0 | 2 |
| Carlsson, F., et al. (2021)^6^ | 3 | 3 | 3 | 3 | 2 | 2 | 3 | 3 | 3 | 3 | 3 | 0 | 2 |
| Carlsson, F., et al (2022)^7^ | 2 | 3 | 2 | 2 | 2 | 2 | 3 | 3 | 2 | 3 | 3 | 0 | 2 |
| Carrero, I., et al. (2021)^8^ | 2 | 3 | 3 | 2 | 1 | 2 | 3 | 2 | 2 | 2 | 2 | 0 | 1 |
| Costanigro, M., et al (2016).^9^ | 3 | 3 | 3 | 3 | 2 | 3 | 2 | 2 | 2 | 3 | 3 | 1 | 0 |
| De Bauw, M., et al. (2022)^10^ | 3 | 3 | 3 | 3 | 2 | 3 | 3 | 3 | 3 | 3 | 3 | 0 | 3 |
| De Bauw et al. (2024)^11^ | 2 | 3 | 3 | 3 | 1 | 3 | 3 | 3 | 1 | 3 | 3 | 0 | 3 |
| De Marchi, E., et al.( 2016)^12^ | 3 | 3 | 2 | 2 | 1 | 3 | 3 | 3 | 2 | 3 | 3 | 0 | 3 |
| de-Magistris, T. et al (2016)^13^ | 2 | 3 | 3 | 3 | 2 | 3 | 3 | 3 | 2 | 2 | 3 | 1 | 1 |
| Delpozo, B., et al (2025)^14^ | 2 | 3 | 2 | 2 | 1 | 3 | 3 | 3 | 2 | 3 | 3 | 0 | 2 |
| Duckworth, J., et al (2022)^15^ | 2 | 3 | 2 | 3 | 1 | 3 | 3 | 3 | 2 | 3 | 3 | 0 | 3 |
| Fernández-Serrano, P., et al. (2022)^16^ | 2 | 3 | 3 | 2 | 1 | 2 | 3 | 2 | 2 | 2 | 3 | 1 | 2 |
| Fretes, G., et al. (2021)^17^ | 2 | 3 | 3 | 2 | 1 | 1 | 3 | 3 | 3 | 0 | 3 | 0 | 2 |
| Hallstein, E. et al (2013)^18^ | 2 | 2 | 2 | 3 | 1 | 2 | 3 | 3 | N/A | 3 | 3 | 1 | 1 |
| Johnston, R. J., et al. (2001)^19^ | 3 | 3 | 3 | 2 | 1 | 2 | 3 | 2 | 2 | 3 | 3 | 1 | 2 |

**Table A2** continues on next page

**Table A2.** Critical appraisal of articles included in the systematic review according to QuADS guidelines (*cont*).

| **First author** | **1 Theory** | **2 Aim** | **3 Research setting** | **4 Study design** | **5 Sampling** | **6 Data collection (rationale)** | **7 Data collection (format)** | **8 Data collection (procedure)** | **9 Recruitment** | **10 Analytic methods** | **11 Method appropriate** | **12 Stakeholder** | **13 Strengths and limitations** |
| --- | --- | --- | --- | --- | --- | --- | --- | --- | --- | --- | --- | --- | --- |
| Jürkenbeck, K., et al (2023)^20^ | 2 | 3 | 2 | 2 | 2 | 2 | 3 | 3 | 3 | 3 | 3 | 0 | 2 |
| Kaczorowska, J., et al. (2019)^21^ | 2 | 3 | 2 | 2 | 2 | 2 | 3 | 2 | 2 | 3 | 3 | 0 | 1 |
| Lamonaca, E., et al. (2022)^22^ | 2 | 3 | 1 | 2 | 2 | 2 | 2 | 1 | 2 | 3 | 2 | 1 | 1 |
| Latip, M., et al (2024)^23^ | 3 | 2 | 2 | 2 | 1 | 2 | 3 | 2 | 1 | 3 | 2 | 0 | 2 |
| Lazzarini, G. A., et al. (2016)^24^ | 3 | 3 | 3 | 3 | 2 | 3 | 2 | 3 | 2 | 3 | 3 | 0 | 3 |
| Maier, M. (2024)^25^ | 2 | 3 | 2 | 2 | 3 | 2 | 3 | 3 | 3 | 3 | 3 | 2 | 2 |
| Marette, S. (2021)^26^ | 2 | 3 | 2 | 2 | 1 | 2 | 3 | 2 | 2 | 3 | 3 | 0 | 2 |
| Nguyen, M., et al. (2018)^27^ | 3 | 3 | 2 | 2 | 2 | 3 | 3 | 2 | 2 | 3 | 3 | 1 | 1 |
| Paffarini, C., et al (2025)^28^ | 2 | 3 | 2 | 2 | 1 | 2 | 3 | 3 | 2 | 3 | 3 | 0 | 2 |
| Pink, A. E., et al. (2022)^29^ | 2 | 3 | 2 | 2 | 1 | 2 | 3 | 2 | 2 | 3 | 3 | 0 | 3 |
| Risius, A., et al. (2017)^30^ | 3 | 3 | 2 | 3 | 1 | 3 | 3 | 3 | 2 | 2 | 3 | 1 | 3 |
| Scozzafava, G., et al. (2020)^31^ | 3 | 3 | 2 | 2 | 1 | 3 | 3 | 3 | 2 | 2 | 3 | 1 | 2 |
| Shaik, S., et al (2024)^32^ | 3 | 3 | 2 | 2 | 1 | 2 | 3 | 3 | 1 | 3 | 3 | 0 | 2 |
| Sun, X., et al. (2024)^33^ | 3 | 3 | 3 | 3 | 1 | 3 | 3 | 3 | 2 | 3 | 3 | 0 | 3 |
| Taillie, L. S., et al. (2021)^34^ | 3 | 3 | 2 | 3 | 1 | 2 | 2 | 3 | 2 | 2 | 3 | 0 | 3 |
| Taillie, L. S., et al (2024)^35^ | 2 | 3 | 2 | 3 | 2 | 3 | 3 | 3 | 2 | 3 | 3 | 0 | 2 |
| Tait, P.; et al. (2016)^36^ | 3 | 3 | 2 | 2 | 1 | 2 | 3 | 2 | 2 | 3 | 3 | 2 | 1 |
| Van der Waal, N. E., et al. (2022)^37^ | 3 | 2 | 3 | 3 | 1 | 2 | 2 | 3 | 2 | 3 | 3 | 1 | 3 |

**Table A2** continues on next page

**Table A2.** Critical appraisal of articles included in the systematic review according to QuADS guidelines (*cont*).

| **First author** | **1 Theory** | **2 Aim** | **3 Research setting** | **4 Study design** | **5 Sampling** | **6 Data collection (rationale)** | **7 Data collection (format)** | **8 Data collection (procedure)** | **9 Recruitment** | **10 Analytic methods** | **11 Method appropriate** | **12 Stakeholder** | **13 Strengths and limitations** |
| --- | --- | --- | --- | --- | --- | --- | --- | --- | --- | --- | --- | --- | --- |
| Vlaeminck, P., et al. (2014)^38^ | 1 | 2 | 2 | 3 | 1 | 3 | 3 | 3 | 2 | 3 | 3 | 0 | 1 |
| Weinrich, R., et al. (2016)^39^ | 3 | 3 | 2 | 2 | 1 | 2 | 2 | 2 | 1 | 1 | 2 | 0 | 1 |
| Weinrich, R., et al (2016)^40^ | 3 | 2 | 3 | 2 | 2 | 3 | 3 | 3 | 2 | 3 | 3 | 0 | 2 |
| Williams, V., et al (2023)^41^ | 2 | 3 | 2 | 2 | 0 | 3 | 3 | 3 | 1 | 3 | 3 | 0 | 2 |

* QuADS criteria: scale from 0 (representing no mention at all or not appropriate methods) to 3 (representing overall criteria fulfilment) (Harrison *et al.*, 2021). N/A = Does not apply

**References Supplementary Material**

1. Apostolidis C, McLeay F. Should we stop meating like this? Reducing meat consumption through substitution. *Food Policy*. 2016;65:74-89. doi:10.1016/j.foodpol.2016.11.002

2. Arrazat L, Chambaron S, Arvisenet G, et al. Traffic-light front-of-pack environmental labelling across food categories triggers more environmentally friendly food choices: a randomised controlled trial in virtual reality supermarket. *Int J Behav Nutr Phys Act*. 2023;20(1):7. doi:10.1186/s12966-023-01410-8

3. Banovic M, Reinders MJ, Claret A, Guerrero L, Krystallis A. A cross-cultural perspective on impact of health and nutrition claims, country-of-origin and eco-label on consumer choice of new aquaculture products. *Food Res Int Ott Ont*. 2019;123:36-47. doi:10.1016/j.foodres.2019.04.031

4. Berden J, Hung Y. Effectiveness of the Eco-score food label: An information experiment combined with Nutri-score label in Belgium. *Appetite*. 2025;204:107759. doi:10.1016/j.appet.2024.107759

5. Büttner V, Gassler B, Teuber R. Does the Eco-Score lead to a halo effect? Influence of a sustainability label on product perceptions and purchase intention. *Food Qual Prefer*. 2024;121:105246. doi:10.1016/j.foodqual.2024.105246

6. Carlsson F, Kataria M, Lampi E, Nyberg E, Sterner T. Red, yellow, or green? Do consumers’ choices of food products depend on the label design? *Eur Rev Agric Econ*. 2022;49(5):1005-1026.

7. Carlsson F, Kataria M, Lampi E. Sustainable food: Can information from food labels make consumers switch to meat substitutes? *Ecol Econ*. 2022;201:107567. doi:10.1016/j.ecolecon.2022.107567

8. Carrero I, Valor C, Díaz E, Labajo V. Designed to Be Noticed: A Reconceptualization of Carbon Food Labels as Warning Labels. *Sustainability*. 2021;13(3):1581. doi:10.3390/su13031581

9. Costanigro M, Deselnicu O, McFadden DT. Product differentiation via corporate social responsibility: consumer priorities and the mediating role of food labels. *Agric Hum Values*. 2016;33(3):597-609. doi:10.1007/s10460-015-9640-9

10. De Bauw M, De La Revilla LS, Poppe V, Matthys C, Vranken L. Digital nudges to stimulate healthy and pro-environmental food choices in E-groceries. *Appetite*. 2022;172:105971. doi:10.1016/j.appet.2022.105971

11. De Bauw M, Peracaula Moner A, Santa Cruz E, Vranken L. Please don’t throw me in the briar patch! Empirical evidence on the role of instructional cues on eco-label usage in fish consumption decisions. *Appetite*. 2024;197:107291. doi:10.1016/j.appet.2024.107291

12. De Marchi E, Caputo V, Nayga R, Banterle A. Time preferences and food choices: Evidence from a choice experiment. *Food Policy*. 2016;62(C):99-109.

13. de-Magistris T, Gracia A. Consumers’ willingness-to-pay for sustainable food products: the case of organically and locally grown almonds in Spain. *J Clean Prod*. 2016;118:97-104. doi:10.1016/j.jclepro.2016.01.050

14. Delpozo B, Pons-Gómez A, Besada C. Eye-tracking study on the impact of ‘EU organic’ and ‘sustainable irrigation’ logos on consumer acceptance of olive oil. *J Sci Food Agric*. 2025;105(3):1864-1874. doi:10.1002/jsfa.13963

15. Duckworth JJ, Randle M, McGale LS, et al. Do front-of-pack ‘green labels’ increase sustainable food choice and willingness-to-pay in U.K. consumers? *J Clean Prod*. 2022;371:133466. doi:10.1016/j.jclepro.2022.133466

16. Fernández-Serrano P, Tarancón P, Bonet L, Besada C. Consumers’ Visual Attention and Choice of ‘Sustainable Irrigation’-Labeled Wine: Logo vs. Text. *Agronomy*. 2022;12(3):685. doi:10.3390/agronomy12030685

17. Fretes G, Sepúlveda A, Corvalán C, Cash SB. Children’s Perceptions about Environmental Sustainability, Food, and Nutrition in Chile: A Qualitative Study. *Int J Environ Res Public Health*. 2021;18(18):9679. doi:10.3390/ijerph18189679

18. Hallstein E, Villas-Boas SB. Can household consumers save the wild fish? Lessons from a sustainable seafood advisory. *J Environ Econ Manag*. 2013;66(1):52-71.

19. Johnston R, Roheim C, Donath H, Asche F. Measuring consumer preferences for ecolabeled seafood: An international comparison. *J Agric Resour Econ*. 2001;26(1). Accessed October 31, 2023. https://econpapers.repec.org/article/agsjlaare/31157.htm

20. Jürkenbeck K. The effect of information among established and new sustainability labelling on consumers’ preference and willingness to pay. *Clean Responsible Consum*. 2023;10:100131. doi:10.1016/j.clrc.2023.100131

21. Kaczorowska J, Rejman K, Halicka E, Szczebyło A, Górska-Warsewicz H. Impact of Food Sustainability Labels on the Perceived Product Value and Price Expectations of Urban Consumers. *Sustainability*. 2019;11(24):7240. doi:10.3390/su11247240

22. Lamonaca E, Cafarelli B, Calculli C, Tricase C. Consumer perception of attributes of organic food in Italy: A CUB model study. *Heliyon*. 2022;8(3):e09007. doi:10.1016/j.heliyon.2022.e09007

23. Muhammad Safuan Abdul Latip, Azlina Samsudin, Malisa Sahila Abdul Manap, Mohd Hazrin Iman Noorkhizan. MODELLING THE SUSTAINABLE CHOICES: THE INFLUENCE OF LABELS AND ATTITUDES ON CONSUMER PURCHASE INTENTIONS OF ORGANIC FOOD. *Int J Bus Soc*. 2024;25(3):1126-1144. doi:10.33736/ijbs.8572.2024

24. Lazzarini GA, Zimmermann J, Visschers VHM, Siegrist M. Does environmental friendliness equal healthiness? Swiss consumers’ perception of protein products. *Appetite*. 2016;105:663-673. doi:10.1016/j.appet.2016.06.038

25. Maier M. Increasing the uptake of plant-based diets: An analysis of the impact of a CO2 food label. *J Environ Psychol*. 2024;93:102216. doi:10.1016/j.jenvp.2023.102216

26. Marette S. Ecological and/or Nutritional Scores for Food Traffic-Lights: Results of an Online Survey Conducted on Pizza in France. *Sustainability*. 2022;14(1):247. doi:10.3390/su14010247

27. Nguyen H. Sustainable food systems: Concept and framework. Published online 2018. https://www.fao.org/3/ca2079en/CA2079EN.pdf

28. Paffarini C, Torquati B, Cecchini L. The impact of multiple labeling on consumer choices for extra virgin olive oil: A cross-country study. *Agric Food Econ*. 2025;13(1):26. doi:10.1186/s40100-025-00363-9

29. Pink AE, Stylianou KS, Ling Lee L, Jolliet O, Cheon BK. The effects of presenting health and environmental impacts of food on consumption intentions. *Food Qual Prefer*. 2022;98:104501. doi:10.1016/j.foodqual.2021.104501

30. Risius A, Janssen M, Hamm U. Consumer preferences for sustainable aquaculture products: Evidence from in-depth interviews, think aloud protocols and choice experiments. *Appetite*. 2017;113:246-254. doi:10.1016/j.appet.2017.02.021

31. Scozzafava G, Gerini F, Boncinelli F, Contini C, Marone E, Casini L. Organic milk preference: is it a matter of information? *Appetite*. 2020;144:104477. doi:10.1016/j.appet.2019.104477

32. Shaikh S, Yamim AP, Werle COC. Are all-encompassing better than one-trait sustainable labels? The influence of Eco-Score and organic labels on food perception and willingness to pay. *Appetite*. 2024;203:107670. doi:10.1016/j.appet.2024.107670

33. Sun X, Wang R, He P, Liu B. Effects of environmental and nutritional labels on the dietary choices of consumers: Evidence from China. *Environ Impact Assess Rev*. 2024;105:107407. doi:10.1016/j.eiar.2023.107407

34. Taillie LS, Busey E, Stoltze FM, Dillman Carpentier FR. Governmental policies to reduce unhealthy food marketing to children. *Nutr Rev*. 2019;77(11):787-816. doi:10.1093/nutrit/nuz021

35. Taillie LS, Wolfson JA, Prestemon CE, et al. The impact of an eco-score label on US consumers’ perceptions of environmental sustainability and intentions to purchase food: A randomized experiment. *PLOS ONE*. 2024;19(6):e0306123. doi:10.1371/journal.pone.0306123

36. Tait P, Saunders C, Guenther M, Rutherford P, Miller S. Exploring the impacts of food label format on consumer willingness to pay for environmental sustainability: A choice experiment approach in the United Kingdom and Japan. *Int Food Res J*. 2016;23(4):1787-1796.

37. van der Waal NE, Folkvord F, Azrout R, Meppelink CS. Can Product Information Steer towards Sustainable and Healthy Food Choices? A Pilot Study in an Online Supermarket. *Int J Environ Res Public Health*. 2022;19(3):1107. doi:10.3390/ijerph19031107

38. Vlaeminck P, Jiang T, Vranken L. Food labeling and eco-friendly consumption: Experimental evidence from a Belgian supermarket. *Ecol Econ*. 2014;108:180-190. doi:10.1016/j.ecolecon.2014.10.019

39. Weinrich R, Franz A, Spiller A. Multi-level labelling: too complex for consumers? *Econ Agro-Aliment*. 2016;18(2):155-172.

40. Weinrich R, Spiller A. Developing food labelling strategies: Multi-level labelling. *J Clean Prod*. 2016;137:1138-1148. doi:10.1016/j.jclepro.2016.07.156

41. Williams V, Flannery O, Patel A. Eco-score labels on meat products: Consumer perceptions and attitudes towards sustainable choices. *Food Qual Prefer*. 2023;111:104973. doi:10.1016/j.foodqual.2023.104973
